# Prevalence of anxiety and its associated factors among infertile patients after ‘two-child’ policy in Chongqing, China: a cross-sectional study

**DOI:** 10.1186/s12978-021-01140-9

**Published:** 2021-09-30

**Authors:** Wenwu Gui, Xi Yang, Huimin Jiang, Hongwen Wu, Mao Zeng, Yidi Wen, Tian Qiu, Yong Zhang, Zhi Ma, Chao Tong, Li Luo, Yong Zhao, Lianlian Wang

**Affiliations:** 1 Department of Reproductive Center, The First Affiliated Hospital of Chongqing Medical University, Youyi Road, Yuzhong, Chongqing, China; 2 School of Public Health and Management, Chongqing Medical University, Yixueyuan Road, Yuzhong, Chongqing, 400016 China; 3 Research Center for Medicine and Social Development, Chongqing Medical University, Chongqing, 400016 China; 4 The Innovation Center for Social Risk Governance in Health, Chongqing Medical University, Chongqing, 400016 China; 5 Department of Psychiatry, The First Affiliated Hospital of Chongqing Medical University, Chongqing, 400016 China; 6 Department of Obstetrics, The First Affiliated Hospital of Chongqing Medical University, Chongqing, 400016 China; 7 Canada-China-New Zealand Joint Laboratory of Maternal and Fetal Medicine, Chongqing Medical University, Chongqing, 400016 China

**Keywords:** Anxiety disorder, Infertility, Two-child policy

## Abstract

**Background:**

With the prevalence of infertility increasing every year around the world, it has seriously impacted the individual quality of family and social life. Anxiety is one of the most prevalent anxiety disorders among infertile patients. After the two-child policy, whether it affected the prevalence of anxiety is controversial. This study aimed to determine the prevalence of anxiety and its potential risk factors among Chinese infertile women after the enforcement of ‘two-child policy’.

**Methods:**

This cross-sectional study included 693 infertile patients in a reproductive medical center in Chongqing, China, between February 2016 and December 2018. Data was collected by Self-filling questionnaires including basic demographic information and the Generalized Anxiety Disorder-7 (GAD-7). SPSS statistical software (IBM SPSS version 25) was used to analyse the obtained data. Descriptive analysis was used to describe basic information and anxiety scores, the chi-square test and binary logistic regression were used to analyse the relationship between anxiety and other variables.

**Results:**

The prevalence of anxiety among total infertile patients was 21.8%, and its 23.5% among first-child infertile patients (FI), and 18.4% among second-child infertile patients (SI) respectively (P > 0.05). Binary logistic regression showed that patients with lower education levels were more likely to have anxiety (P < 0.01). Patients with middle salary incomes were more likely to have anxiety (OR = 1.860, 95% CI: 1.068–3.238). Oral contraception taking history (OR = 1.778, 95% CI: 1.186–2.667), and history of allergy (OR = 2.098, 95% CI: 1.219–3.612) were associated with anxiety.

**Conclusions:**

Under the full liberalization of the “two-child policy”, the total prevalence of anxiety among Chinese infertile female is comparatively high. Low education levels, middle incomes, oral contraception taking and allergy history can be the related risk factors of anxiety. We promote that all infertile patients should be evaluated for the prevalence of anxiety, especially those with potential risks, and receive consultant or targeted treatment when needed.

## Plain language summary

With the formulation of China's two-child policy and the increase in the incidence of infertility, anxiety is one of the most common anxiety disorders among infertility patients. This study mainly discussed the prevalence of anxiety among infertile women in China and its potential risk factors after the implementation of the "two-child" policy.

First-child infertile patients (FI) or Second-child infertile patients (SI) were asked to self-fulfilled the 7-item Generalized Anxiety Disorder scale questionnaires to understand their anxiety.

Of the 693 respondents: the prevalence of anxiety among total infertile patients was 21.8%, and its 23.5% among first-child infertile patients, and 18.4% among second-child infertile patients. The group of infertile patients with middle salary incomes, a history of oral contraceptive use, and a history of allergy were more likely to be anxious.

In conclusion: under the current situation, low education levels, middle incomes, OC taking and allergy history can be the related risk factors of anxiety. It is of guiding significance for the mental health of special populations of infertility.

## Abstract in French

**Contexte:**

La prévalence de l'infertilité augmente chaque année dans le monde, elle a gravement affecté la qualité de vie individuelle, familiale et sociale. L'anxiété est l'un des troubles anxieux les plus répandus chez les patients infertiles. Après la «politique des deux enfants», si elle a affecté la prévalence de l'anxiété est controversée.

Cette étude visait pour déterminer la prévalence de l’anxiété et ses facteurs de risque potentiels chez les femmes chinoises stériles après l’application de la «politique des deux enfants».

**Méthodes:**

Cette étude transversale a inclus 693 patients stériles dans un centre de médecine de la reproduction à Chongqing, Chine, entre février 2016 et décembre 2018. Les données ont été collectées par des questionnaires auto-remplis comprenant des informations démographiques de base et le trouble d'anxiété généralisée-7 (TAG-7). Le logiciel statistique SPSS (IBM SPSS version 25) a été utilisé pour analyser les données obtenues. L'analyse descriptive a été utilisée pour décrire les informations de base et les scores d'anxiété, le test de chi carré et la régression logistique binaire ont été utilisés pour analyser la relation entre l'anxiété et d'autres variables.

**Résultats:**

La prévalence de l'anxiété chez les patients stériles totaux était de 21,8%, de 23,5% chez les patients infertiles du premier enfant (FI) et de 18,4% chez les patients infertiles du deuxième enfant (SI) respectivement (p> 0,05). La régression logistique binaire a montré que les patients à faible niveau d’éducation étaient plus susceptibles de souffrir d'anxiété (p <0,01). Les patients à revenus moyen étaient plus susceptibles d'être anxieux (OR = 1,860, IC à 95%: 1,068–3,238). Les antécédents de prise de contraception orale (OR = 1,778, IC à 95%: 1,186–2,667) et les antécédents d'allergie (OR = 2,098, IC à 95%: 1,219–3,612) étaient associés à l'anxiété.

**Conclusions:**

La prévalence totale de l'anxiété chez les femmes chinoises infertiles est comparativement élevée. En Chine, dans la situation actuelle, les niveaux d'éducation, les revenus salariaux et les antécédents de prise de contraception orale peuvent être les facteurs de risque liés à l'anxiété. Nous concluons que tous les patients stériles devraient être évalués pour l'anxiété, en particulier ceux qui présentent des risques potentiels, et devraient recevoir un consultant ou un traitement en cas de besoin.

## Background

With the prevalence of infertility increasing every year around the world, it has seriously impacted the individual quality of family and social life. Worldwide > 186 million people suffer from infertility, the majority being residents of developing countries [1]. It is a problem concerning 8–12% of married couples in the world, which means one in every seven couples in the western world and one in every four couples in developing countries have difficulties in reproducing [2]. In China, the data from the National Health and Family Planning Commission in 2017 showed that the rate of infertility among proper-aged couples reached 15–20% [3]. Thus, it has caused tremendous attention from the society and it was recognized as a key element of reproductive health and a global public health issue by the World Health Organization (WHO) [4].

There are several causes of female infertility including organic lesion and functional factors. More and more evidence has shown that psychological factors might be the major cause of infertility [5–7]. It was reported that psychological stress can contribute to hypothalamic amenorrhea via reducing the hypothalamic–pituitary–ovarian axis [8]. It also can directly cause general activation of the hypothalamic–pituitary–adrenal axis, which leads to the suppression of GnRH pulse generators [9]. It was reported that those infertile patients had faced with enormous pressure, more than 8% of which coming from family and society, and also their daily work and other aspects [4]. All those factors can finally result in infertility.

Infertility can be a very stressful condition, and women are normally blamed for the cause of this situation [3]. Normally, infertile women are not willing to share their story with other family members or friends which may deteriorate the stressful situation. The inability to reproduce naturally can cause feelings of shame, guilt, and low self-esteem. The inability to reproduce naturally can cause feelings of shame, guilt, and low self-esteem. These negative feelings can affect them in many aspects and shrink the quality of their life [10]. Some studies proposed that psychological distress associated with infertility is comparable to that associated with heart disease, cancer, or human immunodeficiency virus (HIV), and infertile patients undergoing treatment often characterize infertility as the most upsetting experience in their lives [11, 12], which can affect the effectiveness of their therapies. Among those psychological disorders, the most common ones included anxiety [13].

Anxiety is a subjective sense of internal tension, which is often associated with intrusive thoughts or worries [14]. A significant segment of the infertile population is affected by anxiety [15]. Several studies have proved the prevalence of anxiety is higher in infertile patients than the control group [16, 17]. Women showed a decrease in anxiety when treatment successfully resulted in a pregnancy [18]. Although recent studies have shown the importance of anxiety and its negative effect on the infertile treatment outcomes, there are few studies in China about revealing the current situation, its prevalence and associated factors among infertile patients, especially after the enforcement of the new family plan, which is called ‘two-child’ policy. In China, with the enforcement of the second-child policy and the traditional concept of ‘more children, more blessings’, a baby boomlet has been set up to ease the current condition of population ageing, sex ratio unbalance and approximately lowest-low fertility rate in Chinese society.

The consequences of this change are inevitably speculative, meanwhile, there are a series of problems needed to be solved. The aims of the current study were to determine the prevalence of anxiety in infertile patients, and to explore associated factors among these patients. To further improve reproductive capability, caring for female reproductive health in a better way.

## Material and methods

This study was conducted at the Reproductive Medical Center of the First Affiliated Hospital of Chongqing Medical University in Chongqing City over a period of 34 months from February 2016 to December 2018. This study was approved by The Ethics Committee of The First Affiliate Hospital of Chongqing Medical University (No. 2018-131).

All participants had to meet the following criteria (a) willing to participate; (b) age over 18 and under 50; (c) premenopausal; (d) been unsuccessful in conceiving a baby with unprotected intercourse for more than 1 year; (e) ability of Chinese reading and writing.

Sample size calculation was performed by the formula [19] $N = Z_{1 - \alpha /2}^{2} (1 - p)/\varepsilon^{2} p$. Where we took the incidence of anxiety in Chinese infertility patients (P = 0.614) [20]. For a confidence level of 95%, α is 0.05 and *Z*_1−*α*/2_ =1.96. The required sample size N = 242. If we anticipate a 10 ~ 20% rates of the inefficiency, we may want to increase the sample size and total sample size n as 266 ~ 290.

### Data collection tools and procedures

A total of 693 women who fulfilled the 7-item Generalized Anxiety Disorder scale questionnaires were included in the study, and additional patients were excluded. Socio-demographic information including age, educational level, whether a single child in the family, monthly income, oral contraception taking history (OC) and history of allergy were obtained from the respondents. Whether the subjects were presented with first-child infertile patients (FI) or second-child infertile patients (SI) were also acquired. Data relating to the anxiety of infertility was obtained using questionnaire, the 7-item Generalized Anxiety Disorder scale (GAD-7), which has been shown to have satisfactory psychometric properties for infertile people [21]. When we did the survey after all participants were informed of the study and provided written informed consent. Then they completed the questionnaire.

### 7-Item generalized anxiety disorder scale(GAD-7)

The GAD-7 is a 7-item self-report scale. It contained seven questions that have bothered participants during the preceding 2 weeks. It was recommended for evaluating generalized anxiety disorder and its severity. The response options are as follows: not at all; several days; more than half the days; and nearly every day. Higher scores indicate greater generalized anxiety (the total scores range is 0–21). Scores >  = 5 indicates anxiety. The classifications of severity are:

1. 0–4 (without anxiety)
2. 5–9 (mild anxiety)
3. 10–13 (moderate anxiety)
4. 14–18 (moderately severe anxiety)
5. 19–22 (severe anxiety)

### Data processing and analysis

All data were analysed using SPSS version 25 (SPSS Inc., Chicago, IL, USA). Frequency tables were used for the descriptive purposes. Continuous variables were presented as mean ± standard deviation (SD) and categorical variables as numbers (percentage). The relationship between categorical responses and explanatory variables were evaluated using a chi-square test. Binary logistic regression was performed considering anxiety as the dichotomous variable to identify the association between anxiety and demographic or fertility variables. In all statistical tests, a value of P < 0.05 was considered significant.

## Results

### Demographic description of FI and SI patients

The demographic description of FI and SI patients were presented in Table 1. The age of the study population ranged from 24 to 48 years old with a mean ± standard deviation of 35.26 ± 4.30 years. FI was prevalent in 66.2% of the study population, while 33.8% of them had SI. More SI patients are of minor ethnicity than FI patients (P < 0.01). 79.7% of SI patients had an elective abortion, as well as 45.4% in FI patients (P < 0.01). FI patients had higher education levels (undergraduate and above) than SI patients (P < 0.01). 52.5% of FI patients are a single child in the family, while 20.3% of SI patients are a single child in the family (P < 0.01). FI patient made higher monthly income than SI did (P < 0.01).

**Table 1:** Demographic characteristics of initial fertility patients (n = 459) and secondary infertile patients (n = 234)

Variables	First child infertility(n = 459)	Second child infertility(n = 234)	χ^2^	P value
	No. (%)	No. (%)		
*Age*	n = 452	n = 233	68.171	0.000**
20–30	31(6.9)	16(6.9)		
31–40	395(87.4)	151(64.8)		
> 40	26(5.8)	66(28.3)		
*Ethnicity*	n = 453	n = 231	26.637	0.000**
Han	441(97.4)	202(87.4)		
Minority	12(2.6)	29(12.6)		
*Elective abortion*	n = 412	n = 200	58.184	0.000**
Yes	196(47.6)	160(80.0)		
None	216(52.4)	60(20.0)		
*OC history*	n = 429	n = 216	5.581	0.020*
Yes	223(52.0)	91(42.1)		
No	206(48.0)	125(57.9)		
*Education level*	n = 453	n = 231	36.814	0.000**
Junior school and blow	11(2.4)	8(3.5)		
Junior high School	48(10.6)	57(24.7)		
High school	44(9.7)	36(15.6)		
Undergraduate	311(68.7)	122(52.8)		
Graduate and above	39(8.6)	8(3.5)		
*History of allergy*	n = 442	n = 230	0.571	0.450
Yes	59(13.3)	26(11.3)		
No	383(86.7)	204(88.7)		
*Monthly salary*	n = 454	n = 225	13.248	0.001*
Low	123(27.1)	92(40.9)		
Middle	222(48.9)	90(40.0)		
High	109(24.0)	43(19.1)		
*Single child in the family*	n = 422	n = 203	41.392	0.000**
Yes	205(52.5)	44(21.7)		
No	217(47.5)	159(78.3)		
*Anxiety*	n = 459	n = 234	2.415	0.120
Yes	108(23.5)	43(18.4)		
No	351(76.5)	191(81.6)		

*P < .05; **P < .001

### Anxiety results

Levels of anxiety among FI and SI women are listed in Table 1. There is no significant difference between FI and SI patients about the occurrence rate of anxiety (P > 0.05). Of those patients who had FI, 20.9% had mild anxiety; 2.4% had moderate anxiety; 0.2% had moderately severe anxiety and 0% had severe anxiety. To patients with SI, 13.7% had mild anxiety; 1.7% had moderate anxiety; 1.3% had moderately severe anxiety and 1.7% had severe anxiety. Demographic data of anxious and non-anxious patients are listed in Table 2. More than half of anxious patients (59%) had history of taking OC, while the rate was 45.7% among non-anxious patients (P = 0.005). More non-anxious patients had higher education levels than anxious patients (P = 0.003)0.18.6% anxious patients had allergic history, and 11% of non-anxious patients had an allergic history (P = 0.015). There is also significant difference between those two groups about the monthly income (P = 0.020). More anxious patients are in the group of middle incomes (54.0%), and more non-anxious patients (24.6%) had higher incomes than anxious patients (14.7%).

**Table 2:** Demographic and infertile characteristics of anxious (n = 151) and non-anxious (n = 543) patients

Variables	Anxious patients (n = 151)	Non-anxious patients (n = 542)	χ^2^	P value
	No. (%)	No. (%)		
*Age*	n = 452	n = 233	3.597	0.166
20–30	9(6.2)	38(7.1)		
31–40	124(84.9)	422(78.3)		
> 40	13(8.9)	79(14.7)		
*Ethnicity*	n = 146	n = 538	0.474	0.491
Han	139(95.2)	504(93.7)		
Minority	7(4.8)	34(6.3)		
*Elective abortion*	n = 125	n = 487	1.862	0.172
Yes	66(52.8)	290(59.5)		
None	59(47.2)	197(40.5)		
*OC history*	n = 142	n = 503	7.994	0.005*
Yes	84(59.2)	230(45.7)		
No	58(40.8)	273(54.3)		
*Education level*	n = 150	n = 534	16.309	0.003*
Junior school and blow	8(5.3)	11(2.1)		
Junior high School	35(23.3)	70(13.1)		
High school	16(10.7)	64(12.0)		
Undergraduate	85(56.7)	348(65.2)		
Graduate and above	6(4.0)	41(7.7)		
*History of allergy*	n = 145	n = 527	5.968	0.015*
Yes	27(18.6)	58(11.0)		
No	118(81.4)	469(89.0)		
*Monthly salary*	n = 150	n = 529	7.847	0.020*
Low	47(31.3)	168(31.8)		
Middle	81(54.0)	231(43.7)		
High	22(14.7)	130(24.6)		
*Single child in the family*	n = 133	n = 492	0.634	0.426
Yes	49(36.8)	200(40.7)		
No	84(63.2)	292(59.3)		

*P < .05; **P < .001

### Factors associated with anxiety

Binary logistic regression was used to exam the association between anxiety and other variables which are education levels, monthly incomes, OC history and allergic history (Table 3). According to the analysis, patients with lower education levels are more likely to have anxiety (P < 0.01). Patients with middle salary incomes are more likely to have anxiety than those with high incomes (OR = 1.860, 95% CI: 1.068–3.238, P = 0.028), also did patients with low income when compare with high incomes patients, but the difference was not statistically significant (OR = 1.049, 95% CI: 0.553–1.992, P = 0.884). Patients who took OC are 1.778 times more likely to have anxiety than those who didn’t (OR = 1.778, 95% CI: 1.186–2.667, P = 0.005). Patients with allergic history are 2.098 times more likely to have anxiety (OR = 2.098, 95% CI: 1.219–3.612, P = 0.007).

**Table 3:** Association between anxiety and demographic/fertility variables among infertile patients by binary logistic regression model

Variable	Anxiety^a^			
	B-coefficient	P value	OR	95% CI
*Education level*		0.001^**^		
Junior school and blow	1.980	0.008^**^	7.245	1.673–31.363
Junior high School	1.491	0.004^**^	4.439	1.599–5.149
High school	0.563	0.304	1.757	0.599–5.149
Undergraduate	-0.657	0.234	1.75	0.697–4.397
Graduate and above			1.000	
*Monthly salary*		0.017^*^		
Low	0.048	0.884	1.049	0.553–1.992
Middle	0.621	0.028^*^	1.86	1.068–3.238
High			1.000	
*OC history*				
Yes	0.576	0.005^**^	1.778	1.186–2.667
No			1.000	
*History of allergy*				
Yes	0.741	0.007^**^	2.098	1.219–1.612
No			1.000	

*95%CI* 95%Confidence interval, *OR* odds ratio; *P < .05; **P < .001

^a^Adjusted model for anxiety that included age, ethnicity, elective abortion, and whether a single child in the family

## Discussion

According to the demographic information we collected, our research revealed that the prevalence of anxiety is comparatively high. The prevalence of anxiety in infertile patients have been assessed in several countries. Enikő Lakato reported the prevalence of anxiety among infertile patients was 39.6% in Hungary [16]. In Sweden, one study revealed that anxiety was detected in 14.8% of infertile patients in 2008 [22]. A study from the infertility clinics in northern California determined that 76% of the infertile women reported significant symptoms of anxiety [23]. The data from Iran was 56.9% [17]. In China, anxiety was represented in 21.3% of infertile patients [24]. In Japan, researchers used the Japanese version of the State–Trait Anxiety Inventory (STAI) scale and found that infertility patients have a higher incidence of anxiety [25]. Indeed, the incidence of anxiety can vary depending on culture, ethnicity, lifestyle, and religion. However, compared to the data used in our study, the overall incidence of anxiety in infertile patients is still relatively high.

After the full liberalization of the "second child policy", factors such as age, social roles and intricate family problems all contributed to more complex re-fertility nature in patients, thus prompting us to compare these patients. We discovered that the incidence of anxiety in "one-child" infertility patients and "Second-child" infertility patients are high, but there is no significant difference between the two groups. For patients with "second child" infertility, the general public often holds the opinion that they should not be anxious, as the "second child" should be an easier step after “one child”. However, the results of this study found that the incidence of anxiety in patients with "second child" infertility is still high, and there is no statistical difference compared with "one-child" infertility patients. This suggests that whether the infertility patient is from a "one-child" or "second child" background, council and assistance should be offered to examine their psychological status and to provide psychological intervention if necessary.

In our study, among the whole infertile patients, the likelihood of anxiety was increased with lower educational levels, which is in accordance with some previous studies [26, 27]. It is estimated that higher educational patients can have more and easier access to information. They are more open-minded to ‘child issue’, and with a better mindset to accept the negative consequence of being infertile. Patients with high education level are generally higher in social status with multiple hobbies that can alleviate the negative emotions caused by infertility. Patients with low levels of education are more susceptible to traditional thinking and believe that having children is the primary task of life. As such, they do not fully comprehend their condition and situation, which make them even more susceptible to anxiety.

Usually, patients with lower incomes are more likely to suffer from anxiety because of the long treatment period of infertility, high cost, and not within the scope of medical insurance [28]. Dijkstra-Kersten and colleagues’ research proposed that financial strain was related to depressive and/or anxiety disorder, above the effect of income [29]. Interestingly, our study showed that patients with middle salary incomes are more likely to have anxiety than those with high or low incomes. A possible explanation might be due to the small sample size used in the study, which requires further research. Another possible explanation for this occurrence relates to the social status and lifestyle differences between income levels. In China, most patients with low incomes still live at the countryside where the living cost is comparatively low, and their awareness and expectation about life is also low. On the other hand, middle incomes patients live in the city, where the living cost is higher and they are more likely to have financial strain. In addition, the process of diagnosis and treatment of infertility takes long periods, the treatment effect has uncertain factors, and patients often need to take time off to arrange work-time to see a doctor, affecting income.

Oral contraception (OC) history has been found to be associated with the prevalence of anxiety according to our study. Taking OC will cause a higher risk of anxiety. One study including 202 women revealed the relationship between OC and mood [30], which is OC use was related to a small, but statistically significant increases in mean anxiety, and mood swings scores among inter-menstrual phase women (from day 5 of the menstrual cycle to day 21). Another study reported that approximately 4–10% of combined oral contraceptive users made complain of adverse mood symptoms including increased anxiety [31]. One possible explanation for this phenomenon is OC can cause lower endogenous estradiol levels, and estradiol is suggested to be associated with mood improvement [32]. A higher continued vigilance for negative emotional stimuli and a biased attention towards negative stimuli in OC-users, possibly explaining adverse effects on mood [33]. However, it is yet unknown if there is any difference in terms of mental health between the infertile and the general OC users, nonetheless infertility itself is a very stressful condition. If possible, for infertile patients, choosing other contraceptive methods could be more beneficial to their physiological situation.

The logistic regression also suggested that patients with a history of allergy are 2.098 times more likely to have anxiety than those patients without it. Patients with a history of allergies are often associated with abnormalities in the immune system, and immune factors are considered to be one of the causes of infertility. There are some studies have found certain levels of anxiety in patients with drug hypersensitive or intolerance and food allergy [34–38]. People with history of allergy could suffer more than the general population. The stress of managing allergy can be associated with impairments in managing anxiety and mood symptoms [39]. One study suggested that patients with a food allergy, their quality of life improves in some but deteriorates in others during oral immunotherapy [40]. At present, there are few studies on the history of allergies and mental health in infertile patients. The effect infertility has on the mental state of the patient still requires further studies. According to the results of our study, the history of allergies and anxiety do occur in infertile patients. The positive correlation indicates a relevant risk factor for concern.

Our study does contain some degrees of limitations around the collection, relationships and quality of data such as reliance on self-report, geographical bias, and missing data respectively. In addition, there is a selection bias in this study. First, since infertility patients were selected in this study, this part of the population may be more sensitive, leading to more serious anxiety rate; Secondly, the location of this survey did not include infertile patients in other hospitals, which may be influenced by location, economy and other aspects, and there may be selection bias. Therefore, in the follow-up study, we need to add infertile patients in other regional hospitals for comparison, to expand the sample size and reduce the bias. Despite these limitations, our study employed a large sample size to reduce bias and variances. In addition, this study is the first to evaluate the prevalence and associated factors of anxiety among infertile patients in Chongqing, China after the enforcement of ‘two-child’ policy.

## Conclusion

In summary, after the full liberalization of the “two-child policy” in China, the overall prevalence of anxiety is at a relatively high level. The incidence of anxiety in initial fertility patients and secondary infertile patients was similar. Anxiety risk factors were associated significantly with low education level, oral contraceptives, and allergy history. Comprehensively assessing their mental health and performing physiological and psychological interventions based on different anxiety-related factors might have therapeutic benefits for infertility women.

### Strengths

- This study is the first to evaluate the prevalence and associated factors of anxiety among infertile patients in Chongqing, China after the enforcement of ‘two-child’ policy.
- Research that combines policy, disease, and psychology to guide the evaluation of future policies in disease.

### Limitations

- Self-reporting questionnaire, the missing data lead to a decrease in the quality of data.
- A selective group of participants who volunteered their participation but couldn’t follow up.

## Data Availability

The datasets generated and/or analysed during the current study are not publicly available due to research data concerns respondents' privacy but are available from the corresponding author on reasonable request.

## Abbreviations

FI: First-child infertility
SI: Second-child infertility
OR: Odds ratio
SD: Standard deviation
OC: Oral contraception
